# Carcass Composition, Meat Quality, Calpain Activity, Fatty Acid Composition and Ribonucleotide Content in Southern Thai Native Goats and Three-Way Crossbred Goats

**DOI:** 10.3390/foods10061323

**Published:** 2021-06-08

**Authors:** Chanporn Chaosap, Nantana Chauychuwong, Ratchasak Chauychuwong, Chatchai Sriprem, Panneepa Sivapirunthep, Awis Qurni Sazili

**Affiliations:** 1 Department of Agricultural Education, Faculty of Industrial Education and Technology, King Mongkut’s Institute of Technology Ladkrabang, Bangkok 10520, Thailand; panneepas@yahoo.com; 2 Department of Animal Science, Faculty of Agriculture, Rajamangala University of Technology Srivijaya, Nakhon Sri Thammarat 80110, Thailand; nantanachu@yahoo.com (N.C.); rchuychuwong@yahoo.com (R.C.); siam_4403@hotmail.com (C.S.); 3 Department of Animal Science, Faculty of Agriculture, Universiti Putra Malaysia, Serdang 43400, Malaysia; awis@upm.edu.my; 4 Institute of Tropical Agriculture and Food Security, Universiti Putra Malaysia, Serdang 43400, Malaysia

**Keywords:** collagen, calpain, cholesterol, flavor, fatty acid

## Abstract

Carcass composition, meat quality, calpain activity, fatty acid composition, and ribonucleotide content were examined and compared between male Southern Thai native goats (NG, *n* = 10) and three-way crossbred goats (CG, 50% Boer × 25% Saanen × 25% Southern Thai native, *n* = 10). All animals were fed 1.5% body weight of concentrate (16% protein and 70% total digestible nutrients) and fresh Napier grass for 4 months. At the end of raising period, the final weight between the two genotypes was not significantly different. The percentage of carcass, bone, and fat of CG were higher than those of NG. NG presented lower shear force values, insoluble and total collagen content but higher collagen solubility and calpain-1 activity at 24 h postmortem. Additionally, NG meat was high in monounsaturated fatty acids but low in saturated fatty acids. However, the amount of ribonucleotide was no significant difference between two genotypes.

## 1. Introduction

Goats are nowadays considered as among the major meat-producing animals as their meat is becoming more acceptable by consumers [1]. The demand for goat meat remarkably increased in Asia and Africa [2] and is also potentially growing in other geographical regions. In Thailand, the goat population continues to grow and has been recorded as one million heads in year 2019 [3]. Most of the goats are owned by smallholder farmers and raised in the southern region of Thailand. Due to the increase in demand, the number of goats raised primarily for meat production grown by 13.67% in 2019 compared to that in 2018 [3]. The traditional goat production system in Thailand is an extensive grazing system [4]. As the traditional system is insufficient to respond effectively for the growing demand, there is a need for an effectively intensive production system. The improvement of growth performance and feed efficiency in the intensive goat production system is a result of a supplement of concentrates together with using improved meat breeds [5].

The improvement in growth performance and productivity has been accomplished through cross breeding programs by crossing Thai native goats with exotic breeds such as Anglo Nubian, Boer, and Saanen [6]. According to [4], a cross breeding program can improve both carcass and meat quality as three-way crossbreds (50% Boer × 25% Anglo-Nubian × 25% Southern Thai native) showed significantly higher slaughter weight and carcass weight, larger loin eye area, longer carcass length, and more tender meat than two-way crossbreds (50% Anglo Nubian × 50% Southern Thai native) and Southern Thai native goats, at similar age.

Goat meat is a good source of dietary protein for health-conscious consumers as it contains less fat and cholesterol than traditional meats such as chicken, pork, beef, and lamb [7]. In addition, goat meat is a healthier meat choice as it is low in saturated fatty acids but high in unsaturated fatty acids, which are positively correlated with a reduction of cardiovascular risks [8]. Meat flavor influences goat meat acceptability and palatability. The ribonucleotides such as inosine 5′ monophosphate (IMP) and guanosine 5′ monophosphate (GMP) are major umami substances in meat those can intensify and improve meat flavor [9]. Inosine and hypoxanthine, the degradation products of IMP during the aging period, can potentially affect meat flavor. They do not contribute to umami taste as inosine is a tasteless substance and hypoxanthine produces a bitter taste [10].

Consumers consider the tenderness of meat as one of the most important attributes in meat quality. The considerable amount and crosslinking of collagen-containing connective tissue can significantly affect meat tenderness. As the crosslinks between the molecules of collagen increases with the animal’s age, this causes lower solubility of collagen which then results in tougher meat texture. [11]. The other important factor affecting meat tenderness is postmortem proteolysis, and the event mainly involves the endogenous calpain proteolytic system. The Ca^2+^ dependent calpain proteolytic system consists of calpain-1, calpain-2 and their endogenous protein inhibitor named calpastatin [12]. Postmortem proteolysis of muscle is mainly coming from calpain-1 and its activity always accompany by its autolysis. The autolysis of calpain-1 during postmortem proteolysis has been used to demonstrate its former activity [13]. The specific protein inhibitor of these enzymes, calpastatin has been reported as the key factor influencing variability in tenderization rate between species (beef < lamb < pork) [14]. Goat meat had higher shear force value and collagen content, so it tended to be tougher than sheep meat [15]. In addition, goat meat tended to have a darker color and lower acceptance compared to lamb [16].

There is scarce scientific information pertaining to the quality characteristics of Thai native goat meat. Additionally, the conducted studies to compare its quality with the improved commercial goat meat breeds are still limited. Therefore, the objectives of this study were to determine and compare carcass composition, meat quality, collagen content, calpain activity, fatty acid composition, and ribonucleotide content between meat obtained from Southern Thai native goats (NG) and three-way crossbred goats (CG) raised under intensive production system.

## 2. Materials and Methods

### 2.1. Animal Ethics

The Animal Care and Use committee of King Mongkut’s Institute of Technology Ladkrabang approved all animal procedures in this study (Approval number: ACUC-KMITL-RES/2019/010).

### 2.2. Experimental Goats and Slaughtering Procedure

Twenty male goats were selected based on similar initial weight, and the selection was done for Southern Thai native goats (NG, *n* = 10) and three-way crossbred goats (CG, 50% Boer × 25% Saanen × 25% Southern Thai native, *n* = 10). They were fed ad libitum amount of fresh Napier grass and 1.5% body weight of concentrate that consisted of 16% protein and 70% Total Digestible Nutrients (TDN), twice daily. The animals were allowed to access unlimited amount of drinking water, throughout the day. The period of adjustment to the feed was 15 days, following which, the animals were fattened for 4 months. The goats were slaughtered after fattening period and carcass physical composition data were obtained.

### 2.3. Muscle Sampling

*Longissimus thoracis* (LT) muscles were taken from the left carcasses within 1 h postmortem. Muscle samples were cut into small pieces, snap-frozen in liquid nitrogen, and stored at ×80 °C for further measurement of calpain activity, and ribonucleotide content. The 1.5 cm thick chop removed from left LT muscle was used to measure drip loss. LT muscles were removed from the right carcasses, measured muscle pH at 45 h postmortem, divided into two of 1-inch-thick steaks, and then kept at 1 °C for 24 h. The first steak was used for pH and color measurement at 24 h postmortem then vacuum-packed, continued up to 7 days at 1 °C and kept at ×20 °C for further cooking loss and shear force measurement. The second steak was vacuum-packed then kept at ×20 °C to further analyze collagen content and fatty acid composition.

### 2.4. Meat Characteristics

#### 2.4.1. pH Measurement

Muscle pH was directly measured at LT muscle steaks as mentioned in 2.3 using a portable pH meter equipped with a spear tip glass electrode (SG2-ELK Seven Go™, Mettler Toledo International Inc., Giessen, Germany).

#### 2.4.2. Color Measurement

The CIE L*, a*, and b* value of three measuring positions of the cut surface of 1-inch-thick LT steak at 24 h postmortem as mentioned in 2.3 after 30 min of blooming at 25 ± 2 °C were measured using a hand-held colorimeter with illuminant D65 and 8 mm of aperture (CR-400 Chromameter, Konica Minolta Sensing Co., Ltd., Tokyo, Japan).

#### 2.4.3. Drip Loss Measurement

Each meat sample was individually weighed, hung in a tightly sealed plastic bag, and stored at approximately 1 °C for 48 h. Meat samples were then cleaned for the excess of superficial juice and reweighed. Drip loss was calculated as a percentage of weight loss before and after suspension.

#### 2.4.4. Cooking Loss and Shear Force Measurement

To measure shear force, the frozen 1-inch-thick steak of each LT muscle was thawed and cooked in an 80 °C water bath until a core temperature of 70 °C was reached. Cooked meat sample was reweighed after cooling down to room temperature. Cooking loss was expressed as a percentage of weight loss during cooking relative to the original weight. Subsequently, the cooked sample was cut paralleled with the muscle fibers direction into eight slices that were 3 cm in length and 1 cm in diameter. The texture analyzer (Model EZ-SX, Shimadzu, Kyoto, Japan) set with a load cell of 50 kg and a crosshead speed of 50 mm/min was used to measure shear force.

### 2.5. Fatty Acid Analysis

The composition of fatty acids (FAs) of LT muscle was determined by gas chromatograph (7890B Agilent, Santa Clara, CA, USA) according to the method previously described [17]. Briefly, the total lipids were extracted from muscle sample by homogenizing with chloroform-methanol (2:1, *v*/*v*) and then evaporating the solvent. The fatty acid methyl esters (FAMEs) were prepared by combing the extracted lipid with 0.5 N Sodium hydroxide/Methanol and internal standard methyl nonadecanoate (SFA-013N, Accu Standard, New Haven, CT, USA), methylated with 10% BF_3_ in methanol, and then analyzed FAs using GC. FAs were identified by comparison of their retention times with those of known standards (F.A.M.E. Mix, C4-C24, Supelco, Bellefonte, PA, USA), and the results was presented as mg/100 g of fresh muscle.

### 2.6. Cholesterol Analysis

Cholesterol determination was carried out following the method described by [17]. Briefly, the pulverized muscle sample was homogenized in saponification reagent (ethanol: 33% KOH (*w*/*v*): 20% ascorbic acid (94:6:0.5)), mixed with HPLC water and hexane, and allowed the sample to separate into phase for 15 h. The non-saponifiable fraction was carefully collected and evaporated with a stream of nitrogen. After dissolving the dry sample with ethyl acetate, cholesterol content was determined by using gas chromatography (7890B Agilent, Santa Clara, CA, USA) equipped with a Zebron ZB-5 column (Phenomenex, Torrance, CA, USA). The mobile phase consisted of a mixture of acetonitrile and isopropanol (70:30, *v*/*v*) with a 0.5 mL/min flow rate. Cholesterol was identified by comparing the retention time of sample peak to that of pure cholesterol standard (Cholesterol C8667, Sigma-Aldrich, Darmstadt, Germany). Cholesterol was quantified by using 5α-cholestane as internal standard.

### 2.7. Ribonucleotide Analysis

The content of ribonucleotide, IMP, GMP, inosine, and hypoxanthine was determined according to the method described by [17]. Briefly, 1 g of pulverized sample was homogenized in 6 mL of cold 0.6 M perchloric acid, left on ice for 15 min, added 5.4 mL of 0.8 M KOH and 0.25 mL of KH_2_PO_4_ buffer, adjusted pH to 7.0 with 0.8 M KOH, and made up to the final volume 15 mL with HPLC water. The mixed sample was centrifuged, and 1 mL of supernatant was collected to analyze the content of ribonucleotide by using the HPLC (Chromaster, Hitachi, Tokyo, Japan).

### 2.8. Collagen Analysis

The collagen analysis was determined according to the method described by [18]. Briefly, the pulverized meat sample of 0.9 g was homogenized in five-fold volume of 1/4-strength Ringer’s solution (0.86% NaCl, 0.03% KCl, and 0.033% CaCl_2_). The homogenate was heated at 77 °C for 60 min in a water bath and then centrifuged at 2500× *g* for 10 min. The supernatant (soluble collagen) and pellet (insoluble collagen) were separated and hydrolyzed for 24 h at 110 °C in 12 N HCL and 6 *n* HCl, respectively. The cool hydrolysate was added with activated carbon, mixed thoroughly, filtered through a rapid filter paper, adjusted pH to approximately 6.7, and diluted the soluble and insoluble fraction with distilled water to either 100 mL or 500 mL, respectively. The diluted hydrolysate was used to measure hydroxyproline concentration. Collagen content was calculated from the hydroxyproline content by multiplying a conversion factor of 7.25.

### 2.9. Casein Zymography

The activities of calpain-1 and calpain-2 were analyzed using casein zymography [19]. Briefly, 0.2 g of pulverized sample was homogenized in 2 mL of extraction buffer [50 mM Tris/HCl (pH 7.5), 5 mM EDTA, 200 μg/mL of 4-(2- aminoethyl)-benzenesulphonyl fluoride, 1 μg/mL of leupeptin, 1 μg/mL of pepstatin]. After centrifugation, the supernatant was taken and mixed with an equal volume of loading buffer [125 mM Tris-HCl pH 6.8, 0.1 M dithiothreitol, 20% (*v*/*v*) glycerol, 0.01% (*w*/*v*) bromophenol blue]. The mixed supernatant was run on native casein acrylamide gel. After electrophoresis, gels were incubated with Ca^2+^ incubation buffer (50 mM Tris-HCl, pH 7.0, 5 mM CaCl_2_, 10 mM DTT), fixed in in 10% (*v*/*v*) acetic acid, stained with Coomassie blue. Band intensities were quantitated by Quantity One Multi Analyst imaging software (Bio-Rad, Hercules, CA, USA).

### 2.10. Statistical Analysis

Data were expressed as mean ± standard deviation. Differences were tested by two-tailed t-test. Pearson’s correlation was used to analyze the association between collagen content, calpain activity, and shear force value parameters. The values *p* < 0.05 were considered statistically significant. Statistical analysis was performed using SAS (SAS Institute Inc., Cary, NC, USA).

## 3. Results

NG and CG with similar initial weight were raised in individual cages for four months and fed with fresh Napier grass and 16% protein concentrate diet at 1.5% of body weight (Table 1). After the raising period there was no significant difference in the final weight between NG and CG (*p* > 0.05). However, CG had higher carcass dressing percentage (*p* < 0.05), and this could be explained by a tendency for them to present heavier carcass weight (*p* = 0.072). For carcass composition, lean percentage was not significantly different between NG and CG. However, CG had higher percentage of bone and fat than those of NG (*p* < 0.01).

The average muscle pH was not significantly different between two genotypes (Table 2). Meat color values of NG and CG were not different. The instrumental color values of L*, a*, and b* in NG and CG meat are 30.01 and 32.77, 13.43 and 13.83, and 13.61 and 14.78, respectively. The average drip loss recorded in the LT muscle from the two genotypes, was 2.07% and 1.93%, for NG and CG, respectively. The percentage of cooking loss was not significantly different between two genotypes. Shear force value of NG was significantly lower than that from CG (*p* < 0.01).

NG had lower amount of insoluble collagen and total collagen content (*p* < 0.05) and higher percentage of collagen solubility (*p* < 0.01, Table 3). The non-denaturing casein zymography was used to measure calpain activity in this study. There was no significant difference in calpain-1 activity at 1 h postmortem (*p* > 0.05) between the two genotypes, while NG showed higher activity of calpain-1 at 24 h postmortem (*p* < 0.05) than that in their counterpart. However, the activity of calpain-2 was not different between the two genotypes (*p* > 0.05). During 24 h postmortem, calpain-1 activity decreased approximately 91% and 92%, while calpain-2 activity decreased 45% and 43% for NG and CG, respectively. The higher decreasing percentage of calpain-1 activity during 24 h postmortem imply greater response of calpain-1 than calpain-2 on postmortem proteolysis.

Pearson’s correlation coefficients between collagen content, calpain activity and shear force value of LT muscle of Southern Thai native goats and three-way crossbred goats are shown in Table 4. Shear force values were not correlated with soluble collagen content and % collagen solubility, while insoluble collagen (r = 0.40, *p* = 0.080) and total collagen content (r = 0.46, *p* = 0.047) were positively correlated with the shear force values. Calpain-1 activity at 1 h postmortem was not correlated (*p* > 0.05), but calpain-1 activity at 24 h postmortem was negatively correlated with shear force values (r = −0.65, *p* = 0.002). The activity of calpain-2 at 1 h and 24 h postmortem was not correlated with shear force (*p* > 0.05).

The content of total cholesterol was recorded as 72 mg/100 g and 80 mg/100 g in NG and CG, respectively, and did not differ between the two genotypes when animals were slaughtered at similar slaughter weight (Table 5). The main individual fatty acids found in the LT muscle of the two genotypes are oleic, palmitic, and stearic acids (Table 5). NG had a significantly higher amount of unsaturated oleic acid (*p* = 007) but a lower amount of saturated myristic acid (*p* = 0.037), palmitic (*p* = 0.027), behenic acid (*p* = 0.011), and stearic acid (*p* = 0.074) leading to higher MUFA (*p* = 0.005) and lower SFA (*p* = 0.001) in NG than those in CG. However, only saturated heneicosylic acid was observed at significantly higher concentration in NG than that in CG (*p* < 0.05). The considerably lower level of saturated palmitic acid and stearic acid in native purebred goats compared to crossbred goats. The desirable fatty acids (DFA), the PUFA/SFA ratio and the ratio of *n*−6/*n*−3 were not affected by goat genotypes (*p* > 0.05).

There was no significant effect of goat genotypes on ribonucleotide content as the amount of IMP, GMP, and inosine did not differ (*p* > 0.05) between NG and CG, with a tendency for the amount of hypoxanthine to be higher in muscles of NG than those of CG (*p* < 0.1, Table 6).

## 4. Discussion

In the present study, the initial weight and final weight of NG and CG were the same, but CG had higher percentage of carcass, bone, and fat than those in NG. However, the lean percentage was not significantly different between two genotypes. According to [6], Southern Thai native goats presented lower body weight at the same age compared to three-way crossbred goats (50% Boer × 25% Anglo-Nubian × 25% Southern Thai native), which were 1.79 and 3.32 kg at birth, 8.10 and 16.02 kg at 3 months old, 12.22 and 25.68 kg at 6 months old, and 16.43 and 34.14 kg at 9 months old, respectively. From the study of [4], male yearlings of three-way crossbred goats (50% Boer × 25% Anglo-Nubian × 25% Southern Thai native) had heavier slaughter weight (37.50 vs. 18.35 kg) and hot carcass weights (19.08 vs. 8.75 kg) and longer carcass length (66.58 vs. 48.42 cm) compared to Southern Thai native goats (*p* < 0.01). However, they reported that hot carcass dressing percentage was not significantly different, between both genotypes, as 48.10% vs. 47.49%, respectively. In contrast, our study found lower hot carcass dressing percentage in NG compared to those of CG at similar slaughter weight with different slaughter age at approximately 9 months old and 6 months old for NG and CG, respectively. The study shows that there was no significant difference on lean percentage between the two genotypes. While NG had lower fat and bone percentage. Anothaisinthawee et al. [4] reported higher bone percentage without any differences in lean or fat percentage between Southern native goat and three-way crossbred goats. Khaokhaikaew et al. [20] also reported higher bone percentage in Anglo-Nubian × Thai native goats with no differences in lean and fat percentages when compared with those of native goats.

The rate and extent of postmortem pH decline has a significant effect on meat quality traits especially to meat color, water holding capacity, and tenderness [21]. In this study, the average muscle pH from both genotypes gradually fell from ~6.5 to ~5.8, over 24 h postmortem (Table 2). The high ultimate pH value in this study records that both NG and CG at 5.97 and 5.74, respectively, is aligned with results from several studies [22,23,24]. A possibility is what [15] and [25] refer to as goats are generally highly prone to stress and the high ultimate pH value is the consequence of preslaughter stress.

The most important quality attribute that influences consumers’ purchasing decision is the color of meat [26]. The usual preference of meat color is bright red meat. Goat meat normally has a darker shade than other red meats [24]. There was no effect of genotype on meat color in this study. The instrumental color values of L*, a*, and b* in both groups were in the range from 30.01 to 32.77, 13.43 to 13.83, and 13.61 to 14.78, respectively. The present results of color values are consistent with those reported in several goat breeds [23,27].

No significant differences in drip loss percentage found in this study was in agreement with [28], who found no significant difference in drip loss percentage between Lat goats and their crossbreds, as 1.31% and 0.93%, respectively. The percentage of cooking loss recorded in the current study falls within the normal range of 27–29% for goat muscles [4,22,24]. Generally, genotypes did not affect water holding capacity, and this was indicated by no significant differences in drip loss and cooking loss percentages between NG and CG. Other studies have not reported that breed affects water holding capacity [22] and cooking loss [4]. Water holding capacity related parameters have been related to the ultimate pH of meat [29]. Therefore, the possible explanation for the similarity of drip loss and cooking loss observed between NG and CG could be explained by the similarity in their ultimate meat pH values.

Meat tenderness is one of the most important quality traits in consumers’ perception to quality of meat. When shear force value of meat is less than 5.5 kg, it can be considered as tender meat [30]. In this study, LT muscles of NG demonstrated lower shear force values than that of CG. However, [4] reported higher shear force values in native goats compared to the three-way crossbred goats. The variation in shear force values between two studies may be attributed to the differences in nutrition, slaughter weight, and age of goats.

The major protein in connective tissue is collagen [31]. The quantity and heat solubility of collagen significantly impact meat tenderness [32]. Tender meat has low collagen content and high collagen solubility [11]. In this study, NG had lower amount of insoluble collagen and total collagen content (*p* < 0.05) and higher percentage of collagen solubility (*p* < 0.01, Table 3). The total collagen content in this study is within the range of 2–6 mg/g, which has been shown in previous studies [33,34].

In this study, the non-denaturing casein zymography was used to measure calpain activity. There was no significant difference in calpain-1 activity at 1 h postmortem (*p* > 0.05) between the two genotypes, while NG showed higher activity of calpain-1 at 24 h postmortem (*p* < 0.05) than that in their counterpart. However, the activity of calpain-2 was not different between the two genotypes (*p* > 0.05). During 24 h postmortem, calpain-1 activity decreased approximately 91% and 92%, while calpain-2 activity decreased 45% and 43% for NG and CG, respectively. The higher decreasing percentage of calpain-1 activity during 24 h postmortem imply greater response of calpain-1 than calpain-2 on postmortem proteolysis. Similarly, [35] reported higher decrease of calpain-1 activity than calpain-2 activity in goat muscle during postmortem storage. Data generated from studies in goats are still limited and thus, the information from other ruminant species such as beef cattle have been used to demonstrate breed effect on the calpain system. A study documented by [36] demonstrated breed effects on the calpain-1 and calpastatin activities. They reported that calpain-1 activity decreased as Brahman percentage increased, and higher calpain-1 activity in 0% Brahman steers than 100% Brahman steers. In addition, earlier, [37] noted that the higher level of calpastatin activity responded for the lower meat tenderness of Brahman purebred compared to *Bos taurus* crossbred.

In this study, the meat of NG was generally more tender than CG meat as it had lower shear force values. The lower amount of collagen content, higher percentage of collagen solubility, and higher activity of calpain-1 at 24 h postmortem could be responsible for greater postmortem tenderization in NG meat than that in CG meat. As expected, there was a positive correlation between shear force and total collagen content and a negative correlation between shear force and calpain-1 activity at 24 h postmortem observed in this study. In agreement with [38] as they reported a positive correlation between shear force and collagen content. The negative relationship between shear force and calpain-1 activity found in this study could be supported by calpain system, especially calpain-1 is responsible for postmortem proteolysis during ageing process resulting in meat tenderization [12,13,14].

The content of total cholesterol was recorded as 72 mg/100 g and 80 mg/100 g in NG and CG, respectively and did not differ between the two genotypes when animals were slaughtered at similar slaughter weight (Table 5). Similarly, [39] reported no genotype effect on cholesterol content. According to these authors, cholesterol values lower than those found in this study, the cholesterol levels from in LT muscle of local Argentina goat and Anglo-Nubian goat ranged from 60–66 mg/100 g. Pratiwi et al. [40] also reported low cholesterol content in LT muscle of castrated Boer goats slaughtered with different ages and weights ranged from 55–60 mg/100 g. However, the values of cholesterol found in this study can be considered moderate as it is lower than 90 mg/100 g [40].

In this study, the main individual fatty acids found in both NG and CG meat are oleic, palmitic, and stearic acids, and this is in agreement with [41,42]. Genotype affected the proportion of the individual fatty acids in this study, consistent with [42] who found lower amount of palmitic acid in Boer than in Australian feral bucks. The considerably lower level of saturated palmitic acid and stearic acid in native purebred goats compared to crossbred goats in this study contradict those reported by [41], that the greater amount of palmitic and stearic acid was recorded in Argentinean native goats than those in their crossbreds.

From a nutritional point of view, NG goats exhibited better nutritional quality according to their meat had higher MUFA and lower SFA compared to CG. Abdullah et al. [43] reported the beneficial health effects of MUFA-rich diets regarding to the improvements of the incidence of coronary heart disease and type 2 diabetes along with the potential economic benefits from healthcare cost savings. The beneficial effects of fatty acids to meat consumers’ needs can be also considered from many aspects such as the DFA, the PUFA/SFA ratio, and the ratio of ω−6: ω−3. The DFA consist of C18:0 and all unsaturated fatty acids and should be of high levels in healthy meat [44].

In general, the PUFA/SFA ratio above 0.45 has been recommended in human diet as a way to prevent coronary heart disease [45]. Within PUFA, *n*-3 PUFA content should be higher than *n*-6 PUFA content since the undesirably higher content of *n*-6 PUFA has been associated with higher incidence of coronary diseases [46]. Earlier, the acceptable value for the *n*-6/*n*-3 ratio was reported as less than 5 [47]. In the current study, the DFA in muscles of NG was slightly higher than those in CG and was recorded as 58.9 g/100 g and 55.6 g/100 g of total fatty acids, respectively. There were no significant differences in PUFA/SFA ratio and *n*−6/*n*−3 ratio between the two genotypes. The PUFA/SFA ratio in muscles of both genotypes ranged between 0.10 and 0.12. The PUFA/SFA ratio recorded by [40] is 0.20 for Australian feral goats at 20 kg slaughter weight. The *n*-6/*n*-3 was comparable to that documented by [39] in Argentinean native Criollo Cordobes goats but was lower than the ratio in Anglo-Nubian goats which are 2.58 and 2.89, and 3.69 and 4.04 at 10 and 11 kg slaughter weight, respectively.

It is well accepted that consumer satisfaction on meat and meat products are highly influenced by tenderness, juiciness, and flavor. Flavor appears to be one of the most important sensory properties in determining consumer acceptability of meat [48]. The ribonucleotides, mainly IMP and GMP contribute to the umami taste in meat and meat products [9]. IMP has been considered as the key index of meat flavor as it is a main component of the flavor of meat [49]. It has been well accepted that ATP in muscle decreases rapidly after animal death, leading to muscle stiffening known as rigor mortis, which is related to a large amount of IMP accumulation [50]. The breakdown products of IMP during postmortem storage, inosine, and hypoxanthine could also impact meat flavor as they have been reported to have either a bitter or tasteless taste [10,51]. The insignificant effect of genotype on ribonucleotide content in this study implies that the flavor intensity of meat from NG and CG were almost the same.

Generally, it has been well established that cross breeding program can improve growth performance and productivity. The improvement of productivity was manifested in this study as higher carcass percentage in three-way crossbred goats compared to native goats. However, the native goats had better meat quality compared to the crossbreds and this is not in agreement with the previous study [4]. The inconsistent findings between studies may be explained by the differences in breed, age and diet, and environmental factors such as climate, weather, husbandry, and management systems.

## 5. Conclusions

Southern Thai native goats demonstrated more tender, higher MUFA, and lower SFA meat. The more tender meat produced by the Southern Thai native could be explained by lower total collagen and insoluble collagen contents, higher percentage of collagen solubility, and higher activity of calpain-1 at 24 h postmortem. The higher carcass dressing percentage shown by the CG group could be due to the higher fat and bone composition. However, despite being larger in terms of carcass weight and dressing percentage, the amount of lean which is economically referred as saleable meat was similar between NG and CG.

## Figures and Tables

**Table 1:** Live weight, carcass weight, carcass percentage, carcass length, and carcass composition of Southern Thai native goats (NG) and Three-way crossbred goats (CG).

Traits	NG ^1^	CG ^1^	*p*-Value
Initial weight (kg)	11.80 ± 2.18	12.98 ± 1.22	0.154
Live weight (kg)	17.33 ± 2.83	18.12 ± 1.52	0.446
Carcass (kg)	7.24 ± 1.43	8.22 ± 0.68	0.072
Carcass dressing (%)	41.65 ± 3.00	45.52 ± 3.91	0.023
Carcass length (cm)	42.30 ± 2.31	44.90 ± 4.68	0.132
Lean (kg)	5.41 ± 0.74	5.64 ± 0.51	0.262
Bone (kg)	1.32 ± 0.14	1.57 ± 0.08	0.0005
Fat (kg)	0.07 ± 0.09	0.18 ± 0.23	0.0002
Lean (%)	63.69 ± 5.68	62.15 ± 6.54	0.581
Bone (%)	16.26 ± 0.59	18.15 ± 0.48	<0.0001
Fat (%)	1.00 ± 0.49	2.22 ± 0.79	0.0006

^1^ Mean ± SD.

**Table 2:** Meat quality characteristics in *Longissimus thoracis* m. of Southern Thai native goats (NG) and Three-way crossbred goats (CG).

Traits	NG ^1^	CG ^1^	*p*-Value
pH_45_	6.46 ± 0.36	6.55 ± 0.28	0.520
pH_24_	5.97 ± 0.45	5.74 ± 0.13	0.150
L*	30.01 ± 3.03	32.77 ± 5.10	0.160
a*	13.43 ± 1.77	13.83 ± 2.99	0.725
b*	13.62 ± 1.69	14.78 ± 2.69	0.261
Drip loss (%)	2.07 ± 0.38	1.93 ± 0.24	0.350
Cooking loss (%)	29.03 ± 2.64	27.29 ± 3.20	0.202
Shear force (*n*)	32.80 ± 2.80	46.30 ± 4.90	<0.0001

^1^ Mean ± SD.

**Table 3:** Collagen content and calpain activity in *Longissimus thoracis* m. of Southern Thai native goats (NG) and Three-way crossbred goats (CG).

Traits	NG ^1^	CG ^1^	*p*-Value
Collagen content ^2^			
Soluble collagen	0.11 ± 0.04	0.09 ± 0.03	0.211
Insoluble collagen	3.02 ± 0.51	3.67 ± 0.71	0.010
Total collagen	3.50 ± 0.52	4.28 ± 0.75	0.015
Collagen solubility (%) ^3^	3.18 ± 0.98	2.10 ± 0.53	0.006
Calpain activity ^4^			
Calpain-1 _1 h_	1.88 ± 0.14	1.74 ± 0.36	0.247
Calpain-1 _24 h_	0.18 ± 0.02	0.14 ± 0.04	0.017
Calpain-2 _1 h_	1.61 ± 0.18	1.58 ± 0.30	0.799
Calpain-2 _24 h_	0.87 ± 0.09	0.88 ± 0.16	0.994

^1^ Mean ± SD. ^2^ mg/g. ^3^ arbitrary densitometry units/0.2 g of wet weight muscle. ^4^$\;\mathrm{Collagen}\;\mathrm{solubility}\;\left(\%\right)=\frac{\mathrm{soluble}\;\mathrm{collagen}\;\mathrm{content}\;}{\mathrm{total}\;\mathrm{collagen}\;\mathrm{content}}\times \;100$.

**Table 4:** Pearson’s correlation coefficients between collagen content, calpain activity, and shear force value of *Longissimus thoracis* m. of Southern Thai native goats and Three-way crossbred goats.

Trait	Pearson’s Correlation Coefficient	*p*-Value
Collagen content		
Soluble collagen	−0.003	0.991
Insoluble collagen	0.401	0.080
Total collagen	0.461	0.047
Collagen solubility (%)	−0.216	0.373
Calpain activity		
Calpain-1 _1 h_	−0.208	0.379
Calpain-1 _24 h_	−0.650	0.002
Calpain-2 _1 h_	−0.082	0.731
Calpain-2 _24 h_	−0.060	0.801

**Table 5:** Cholesterol content and fatty acid composition in *Longissimus thoracis* m. of Southern Thai native goats (NG) and Three-way crossbred goats (CG).

Traits		NG ^1^	CG ^1^	*p*-Value
Cholesterol ^2^		71.88 ± 15.15	79.99 ± 11.38	0.178
Fatty acid composition ^3^				
Myristic acid	C14:0	1.63 ± 0.18	2.29 ± 0.65	0.037
Pentadecanoic acid	C15:0	2.32 ± 0.38	3.08 ± 1.01	0.143
Palmitic acid	C16:0	17.89 ± 1.10	20.08 ± 1.64	0.027
Palmitoleic acid	C16:1	1.80 ± 0.43	1.80 ± 0.44	0.989
Margaric acid	C17:0	13.86 ± 2.11	12.34 ± 2.17	0.253
Heptadecenoic acid	C17:1	1.24 ± 0.14	1.08 ± 0.18	0.114
Stearic acid	C18:0	12.63 ± 1.33	14.32 ± 1.53	0.074
Elaidic acid	C18:1n9t	0.76 ± 0.07	0.81 ± 0.18	0.602
Oleic acid	C18:1n9c	37.00 ± 1.99	30.61 ± 3.84	0.007
Linoleic acid	C18:2n6c	3.72 ± 0.91	4.67 ± 1.44	0.229
α-Linolenic acid	C18:3n3	0.51 ± 0.09	0.44 ± 0.14	0.344
Heneicosylic acid	C21:0	0.46 ± 0.06	0.36 ± 0.07	0.036
Behenic acid	C22:0	0.60 ± 0.27	1.20 ± 0.36	0.011
Tricosylic acid	C23:0	4.36 ± 1.17	5.04 ± 1.98	0.512
Eicosapentaenoic acid	C20:5n3	1.22 ± 0.49	1.88 ± 0.98	0.195
SFA		53.76 ± 1.93	58.73 ± 1.91	0.001
MUFA		40.80 ± 2.20	34.29 ± 3.62	0.005
PUFA		5.44 ± 1.36	6.98 ± 2.39	0.226
DFA		58.87 ± 1.02	55.60 ± 2.42	0.114
PUFA: SFA		0.10 ± 0.03	0.12 ± 0.04	0.423
ω−6		3.72 ± 0.91	4.66 ± 1.44	0.229
ω−3		1.72 ± 0.56	2.32 ± 1.07	0.283
ω−6: ω−3		2.24 ± 0.49	2.32 ± 1.04	0.889

^1^ Mean ± SD. ^2^ mg/100 g. ^3^ g/100g of total fatty acids. SFA (saturated fatty acids): C14:0+C15:0+C16:0+C17:0+C18:0+C21:0+C22:0+C23:0; MUFA (monounsaturated fatty acids): C16:1 + C17:1 + C18:1n9t + C18:1n9c; PUFA (polyunsaturated fatty acids): C18:2n6c + C18:3n3 + C20:5n3;.DFA (desirable fatty acids): MUFA + PUFA + C18:0; ω−6: C18:2n6c; ω−3: C18:3n3+C20:5n3.

**Table 6:** The ribonucleotides content in *Longissimus thoracis* m. of Southern Thai native goats (NG) and Three-way crossbred goats (CG).

Ribonucleotide Content ^1^	NG ^2^	CG ^2^	*p*-Value
Hypoxanthine	0.74 ± 0.28	0.52 ± 0.12	0.091
Inosine	5.44 ± 1.35	5.49 ± 2.21	0.959
Inosine monophosphate	25.61 ± 4.67	28.91 ± 6.58	0.328
Guanosine monophosphate	0.82 ± 0.19	0.88 ± 0.29	0.682

^1^ mg/100 g. ^2^ Mean ± SD.

## Data Availability

The data presented in this study are available on request from the corresponding author.
